# Preparation of Inverse-Loaded MWCNTs@Fe_2_O_3_ Composites and Their Impact on Glycidyl Azide Polymer-Based Energetic Thermoplastic Elastomer

**DOI:** 10.3390/polym17152080

**Published:** 2025-07-30

**Authors:** Shuo Pang, Yihao Lv, Shuxia Liu, Chao Sang, Bixin Jin, Yunjun Luo

**Affiliations:** 1 Shandong Provincial Key Laboratory of Monocrystalline Silicon Semiconductor Materials and Technology, Dezhou University, Dezhou 253023, China; 2 School of Chemistry and Chemical Engineering, Dezhou University, Dezhou 253023, China; 3 Shandong Provincial Engineering Research Center of Organic Functional Materials and Green Low-Carbon Technology, Dezhou 253023, China; 4 School of Materials Science and Technology, Beijing Institute of Technology, Beijing 100086, China

**Keywords:** carbon nanotubes, thermal decomposition, GAP-ETPE, propellant

## Abstract

As a novel carbon material, multi-walled carbon nanotubes (MWCNTs) have attracted significant research interest in energetic applications due to their high aspect ratio and exceptional physicochemical properties. However, their inherent structural characteristics and poor dispersion severely limit their practical utilization in solid propellant formulations. To address these challenges, this study developed an innovative reverse-engineering strategy that precisely confines MWCNTs within a three-dimensional Fe_2_O_3_ gel framework through a controllable sol-gel process followed by low-temperature calcination. This advanced material architecture not only overcomes the traditional limitations of MWCNTs but also creates abundant Fe-C interfacial sites that synergistically catalyze the thermal decomposition of glycidyl azide polymer-based energetic thermoplastic elastomer (GAP-ETPE). Systematic characterization reveals that the MWCNTs@Fe_2_O_3_ nanocomposite delivers exceptional catalytic performance for azido group decomposition, achieving a >200% enhancement in decomposition rate compared to physical mixtures while simultaneously improving the mechanical strength of GAP-ETPE-based propellants by 15–20%. More importantly, this work provides fundamental insights into the rational design of advanced carbon-based nanocomposites for next-generation energetic materials, opening new avenues for the application of nanocarbons in propulsion systems.

## 1. Introduction

Energetic thermoplastic elastomers (ETPEs) refer to a class of thermoplastic elastomers containing energetic groups such as nitrate ester (–ONO_2_), nitro (–NO_2_), nitramine (–NNO_2_), azide (–N_3_), and difluoroamino (–NF_2_) [1]. As a novel polymeric binder, ETPEs have demonstrated great potential in solid propellant applications due to their unique combination of the mechanical properties of thermoplastic elastomers and the energy characteristics of energetic materials. They can provide propellants with high energy density, low sensitivity, reduced signature, and recyclability [2]. Compared with traditional composite solid propellants and double-base propellants, ETPE-based propellants not only achieve a balance between energy and mechanical performance through molecular design but also avoid the processing difficulties caused by high filler loading in composite propellants and the low-temperature brittleness of double-base propellants.

Among various ETPEs, glycidyl azide polyether-based ETPE (GAP-ETPE) has attracted significant attention due to the presence of energetic azide (–N_3_) groups in its backbone [3]. The thermal decomposition of azide groups releases a large amount of nitrogen gas (N_2_) and heat, while the generated nitrogen oxides (NO_x_) can catalyze the decomposition of other propellant components (e.g., ammonium perchlorate and nitramine explosives), thereby significantly enhancing combustion efficiency [4]. GAP-ETPE possesses a segmented structure, where the soft segments (partially or entirely composed of GAP) provide high energy, increasing the burning rate of the propellant, while the hard segments (typically polyurethane-based) contribute to high tensile strength and elastic modulus. As a result, GAP-ETPE propellants have become a research hotspot in the field of thermoplastic elastomer propellants [5].

The thermal decomposition behavior of binders plays a crucial role in determining the combustion performance of solid propellants. In recent years, extensive research has been conducted on the thermal decomposition of GAP-ETPE, with a particular focus on catalytic regulation to optimize its decomposition kinetics [6,7]. Therefore, developing efficient catalysts to tailor the thermal decomposition of GAP-ETPE is of great scientific significance for improving propellant combustion performance.

Multi-walled carbon nanotubes (MWCNTs) have been widely explored as catalyst supports in solid propellants due to their unique tubular structure, high surface area, and excellent electron conductivity. Our previous studies (see Supplementary Materials) revealed that MWCNTs possess stronger catalytic activity than other carbon materials in promoting the thermal decomposition of GAP-ETPE and AP (ammonium perchlorate, a standard oxidizer for solid propellants). MWCNTs are typically loaded with metal oxides (e.g., CuO, Fe_2_O_3_) or nanoparticles to enhance combustion catalysis [8,9,10,11,12]. However, the high aspect ratio of MWCNTs leads to severe entanglement during propellant processing, drastically increasing slurry viscosity and complicating propellant molding. Additionally, exposed nanotube ends act as stress concentration points, promoting crack initiation under mechanical loads and reducing the mechanical properties of the propellant. Furthermore, these exposed ends may form “hot spots” upon external stimuli, increasing the sensitivity and safety risks of the propellant [13,14]. Exposed carbon nanotubes also pose environmental and health risks, for example, in 2020 the International Agency for Chemical Safety (IPCS) added carbon nanotubes to the SIN list (a list of chemicals that should be replaced), mainly due to their carcinogenicity, reproductive toxicity, and environmental persistence. Carbon nanotubes are difficult to degrade in the natural environment and may accumulate through water bodies and soils, causing damage to the nervous system and organs of aquatic organisms such as fish. Thus, addressing the entanglement and exposed ends of MWCNTs is critical for their practical application in solid propellants. Preventing environmental exposure to carbon nanotubes remains a key challenge in industrial production.

To overcome these challenges, this study proposes an inverse design strategy by constructing an MWCNTs@Fe_2_O_3_ composite, which avoids the issues associated with conventional catalyst supports while leveraging the intrinsic catalytic potential of MWCNTs and the synergistic effects of Fe_2_O_3_. The systematic investigation of this composite’s influence on the thermal decomposition of GAP-ETPE will not only provide new insights into the catalytic mechanisms of energetic binders but also lay an experimental foundation for propellant formulation design and the practical application of carbon nanotubes in solid propellants.

## 2. Materials and Methods

### 2.1. Chemicals

The preparation of glycidyl azide polymer energetic thermoplastic elastomer (GAP-ETPE) was achieved by the method reported in the literature [15]. The chain extender was CBA (whose -CN group gives GAP-ETPE molecular bonding function) and BDO. The number-average molecular weight (Mn) of GAP-ETPE was about 29,800 g·mol^−1^, the ratio of hard segment and soft segment was 3:7.

Analytical grade iron nitrate nonahydrate (Fe(NO_3_)_3_·9H_2_O), absolute ethanol, 1,2-propylene oxide, tetrahydrofuran, and hexyl hydride were purchased from Beijing Tong Guang Fine Chemicals Company, Beijing, China and used as-received without further purification. Multi-walled carbon nanotubes (MWCNTs) were purchased from Nanjing XFNANO Materials Tech Co., Ltd., Nanjing, China. RDX (Hexogen), and AP (ammonium perchlorate) and Al (aluminum powder) were provided by Shanxi Beifang Xing’an Chemical Industry Co., Ltd., Taiyuan, China.

### 2.2. Preparation of Samples

(1) Preparation of MWCNTs@Fe_2_O_3_

MWCNTs@Fe_2_O_3_ was prepared by the sol-gel method reported in the reference [6,16]. Fe(NO_3_)_3_·9H_2_O was dissolved in ethanol by stirring until it was completely dissolved. A stoichiometric amount of multi-walled carbon nanotubes (MWCNTs) was then added to the solution. The mixture was ultrasonicated for 30 min to achieve homogeneous dispersion of MWCNTs. Subsequently, propylene oxide was added dropwise under continuous magnetic stirring until gelation occurred. The resulting gel was aged for 72 h, washed thoroughly with ethanol, and dried in a vacuum oven at 120 °C. The resulting solid product was manually ground using an agate mortar to obtain a fine powder, followed by calcination at 300 °C. The composites with MWCNT mass ratios of 1%, 2%, 4%, and 8% relative to Fe_2_O_3_ were labeled as M@F-1, M@F-2, M@F-4, and M@F-8, respectively. For comparison, pure Fe_2_O_3_ (synthesized without MWCNTs) was designated as F, while pristine MWCNTs (untreated) were labeled as M.

Specifically, as a control experiment, we dispersed a predetermined mass of F and MWCNTs (accounting for 1 wt% of F) in n-hexane. The mixture was ultrasonicated for 30 min to ensure homogeneous dispersion, followed by vacuum drying at 80 °C to obtain the solid sample, which was labeled as M+F.

(2) Preparation of MWCNTs@Fe_2_O_3_/GAP-ETPE

Initially, GAP-ETPE was completely dissolved in tetrahydrofuran to form a homogeneous solution. To this solution, 1% by weight of MWCNTs@Fe_2_O_3_ powder was added and uniformly dispersed through 30 min of ultrasonication. Solvent elimination was achieved through 6-hour vacuum drying at 60 °C. The dried composite was then subjected to mechanical mixing in an open mill. The various formulations containing different catalysts (F, M@F-1 through M@F-8, M, and M+F) were systematically labeled from E-F, E-1 to E-8, E-M and E-M+F, while the uncatalyzed reference sample was identified as ETPE. The nomenclature of different catalysts and corresponding GAP-ETPE composites containing these catalysts are summarized in Table 1.

(3) Preparation of MWCNTs@Fe_2_O_3_/GAP-ETPE-Based Propellants

The preparation of propellants followed a similar procedure to that of MWCNTs@Fe_2_O_3_/GAP-ETPE composites. In addition to the catalyst, RDX, Al, and AP were incorporated into the GAP-ETPE solution. The propellant formulation employed in this study has been pre-optimized by our research group. The specific formulations and corresponding designations of the propellants are presented in Table 2. Notably, the determination of 2.4% as the total catalyst concentration was derived from our previous systematic optimization studies.

### 2.3. Measurements and Characterizations

The morphology of the MWCNTs@Fe_2_O_3_ composites was characterized using a field emission scanning electron microscope (SEM, Merlin Compact, Carl Zeiss Microscopy GmbH, Jena, Germany). The thermal performance of the samples was monitored by thermogravimetric-differential scanning calorimetry (TGA-DSC) using a METTLER TOLEDO TGA/DSC 3+ Thermogravimetric Analyzer, Kanton Zürich, Switzerland. The GAP-ETPE/catalysts and propellant samples, placed in an uncovered alumina ceramic crucible, were, respectively, heated from 30 °C to 600 °C at the heating rate of 10 °C min^−1^ under an ultrapure nitrogen atmosphere. The mechanical properties of the propellant were measured using an Instron 68TM-30 universal testing machine. Fracture surface morphology of propellants were characterized by a Hitachi TM4000 SEM (Hitachi High-Technologies Corporation, Tokyo, Japan), while density measurements were performed using a Micromeritics Accupyc II 1345 gas pycnometer, Norcross, GA, USA.

## 3. Results and Discussion

### 3.1. Morphological Characterization of MWCNTs@Fe_2_O_3_ Composites

**Figure 1:**
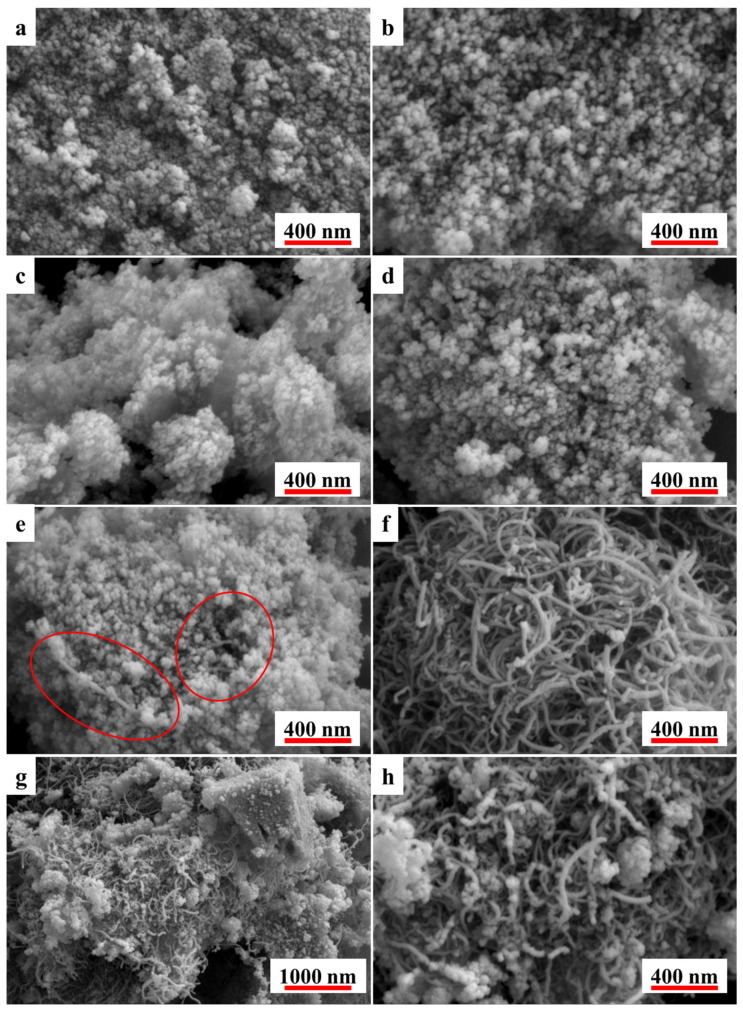
SEM images of F (**a**), M@F-1~M@F-8 (**b**–**e**), M (**f**), and M+F (**g**,**h**). In (**e**), the exposed MWCNTs are marked with red circles.

Surface morphology of the MWCNTs@Fe_2_O_3_ composites were observed by SEM as shown in Figure 1.

Figure 1a reveals that the synthesized Fe_2_O_3_ consists of agglomerates formed by nanoparticles with narrow size distribution (several nanometers in diameter), exhibiting a porous architecture. This observation agrees well with previous reports, confirming the successful preparation of mesoporous Fe_2_O_3_ with structural characteristics consistent with the literature [17].

Figure 1a–e demonstrate that the introduction of MWCNTs does not significantly alter the morphology of Fe_2_O_3_. This observation confirms that the MWCNTs were successfully embedded within the iron oxide gel matrix as designed, demonstrating the successful preparation of the MWCNTs@Fe_2_O_3_ composite material. These findings are further supported by XRD characterization results presented in the Supplementary Materials (Figure S5). Notably, except for the clearly exposed MWCNTs observed in Figure 1e, all other MWCNTs@Fe_2_O_3_ samples show no visible MWCNTs on their surfaces. This indicates effective encapsulation of MWCNTs within the Fe_2_O_3_ gel matrix, perfectly matching our design concept. Such architecture not only incorporates catalytic MWCNTs into the composite but also prevents the aforementioned issues associated with surface-exposed MWCNTs. However, when the MWCNT content exceeds 8 wt%, the Fe_2_O_3_ framework cannot fully accommodate all MWCNTs, resulting in partial exposure (highlighted by red circles in Figure 1e).

Particularly, comparative analysis with Figure 1g–h shows that even at low loading (1 wt%), simple mechanical mixing leads to significant CNT exposure.

### 3.2. Thermal Analysis of MWCNTs@Fe_2_O_3_/GAP-ETPE Composites

As the binder for solid propellants, the thermal decomposition behavior of GAP-ETPE directly influences the combustion performance of the propellant system. Notably, the azide groups (-N_3_) in the GAP-ETPE backbone exhibit a relatively lower decomposition temperature compared to other propellant components, while simultaneously releasing substantial heat during decomposition. Furthermore, the nitrogen-containing compounds generated from this decomposition process can catalytically enhance the decomposition of other propellant constituents. These characteristics make the investigation of its thermal decomposition behavior practically significant for propellant applications. Effects of MWCNTs@Fe_2_O_3_ composites on the thermal decomposition of GAP-ETPE were investigated by TGA and DSC Technique.

**Figure 2:**
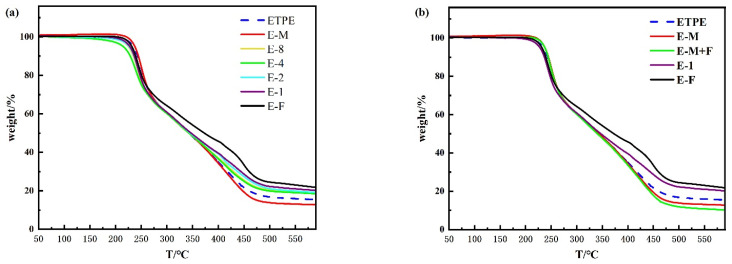

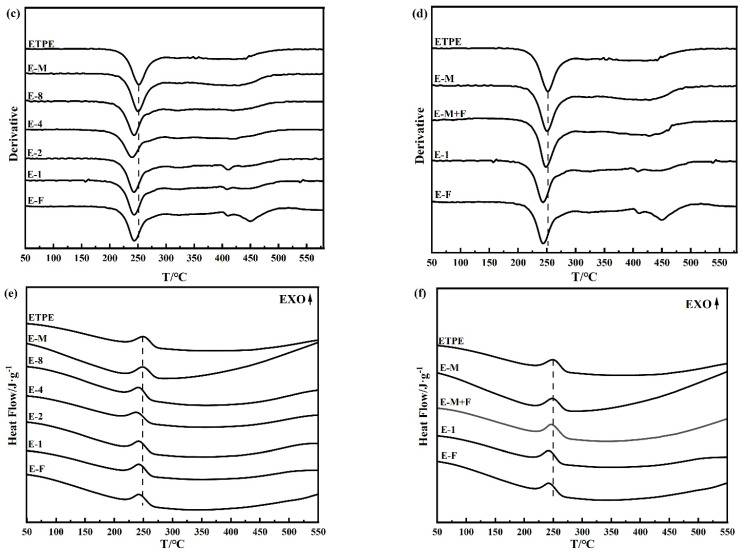
TGA (**a**,**b**), DTG (**c**,**d**), and DSC (**e**,**f**) curves of MWCNTs@Fe_2_O_3_/GAP-ETPE.

Figure 2 presents the thermogravimetric (TGA) and derivative thermogravimetric (DTG) curves of the MWCNTs@Fe_2_O_3_/GAP-ETPE composites, with corresponding quantitative parameters (*T*_p1_—the temperature corresponding to the first DTG peak, *T*_p_—the temperature corresponding to the DSC exothermic peak) summarized in Table 3. Differential scanning calorimetry (DSC) was employed to characterize the thermal effects associated with different decomposition stages of GAP-ETPE, as illustrated in Figure 2 and tabulated in Table 3. For comparative analysis, the thermal behavior of pristine GAP-ETPE is included in all corresponding figures and tables. For clear comparison of catalytic performance across MWCNT-loaded composites and between different catalytic materials, the thermal analysis profiles (TGA/DTG/DSC) are presented in two separate figures.

Figure 2a,b reveal that all samples exhibit three distinct weight loss stages, corresponding to the three decomposition processes of GAP-ETPE [18]. The first weight loss stage occurs between 200 and 290 °C with a mass loss of approximately 30%, which closely matches the theoretical azide group content (29.7%) in GAP-ETPE. Notably, the peak temperature (*T*_p1_) observed in the DTG curve aligns with the exothermic peak temperature (*T*_p_) in the DSC curve, confirming that this initial stage corresponds to azide group decomposition. The second decomposition stage appears in the range of 280–380 °C, attributable to the thermal degradation of urethane segments (hard segments) formed by HMDI, CBA, and BDO. Subsequently, the third weight loss occurs between 380 and 490 °C, which is associated with the decomposition of the polyether backbone, consistent with previous reports [15].

**Figure 3:**
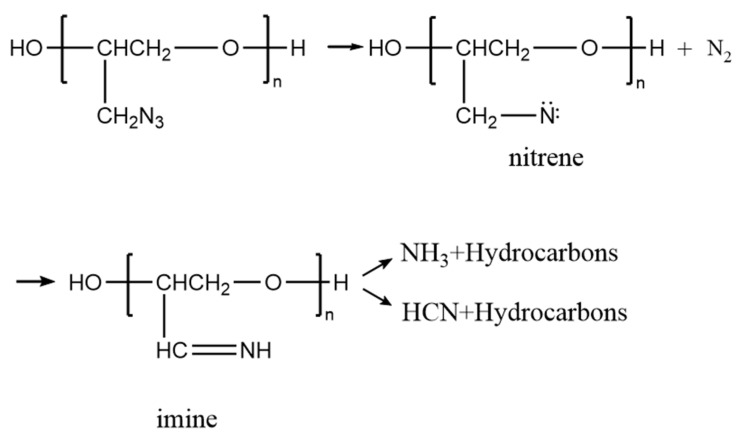
Thermal decomposition process of GAP-ETPE.

The experimental decomposition profile of GAP-ETPE obtained in this study shows perfect consistency with its theoretical decomposition process (Figure 3). The thermal degradation mechanism of azide functional groups initiates with the cleavage of the RN-N_2_ bond, producing reactive nitrene intermediates. Subsequently, these nitrene species undergo nitrogen rearrangement to form imine structures while liberating N_2_ gas. The imine products may then participate in hydrogen transfer and radical-mediated processes to yield NH_3_, or alternatively undergo C-C bond scission to produce HCN. Notably, the generation of NH_3_ is accompanied by heat release (exothermic), whereas HCN formation requires energy input (endothermic) [19].

The residual mass of GAP-ETPE after decomposition varies with different catalysts, primarily due to their distinct adsorption capacities. Materials with mesoporous structures exhibit enhanced adsorption capacity. The Fe_2_O_3_ featuring a gel matrix structure represents a classic type of mesoporous material, demonstrating exceptional adsorption performance. Catalysts with superior adsorption performance tend to retain more decomposition products, resulting in higher measured residues. Specifically, pure Fe_2_O_3_ (F) possesses a mesoporous structure with an exceptionally high specific surface area, exhibiting optimal adsorption characteristics. This explains why sample E-F shows the highest residue content. As the MWCNT content increases in MWCNTs@Fe_2_O_3_ composites (from E-1 to E-8), the carbon nanotubes progressively occupy the pores of Fe_2_O_3_, thereby reducing its adsorption capacity and consequently leading to gradually decreasing char residues. Notably, samples E-M and E-M+F demonstrate even lower residue contents, which may be attributed to the exposed MWCNTs exhibiting higher catalytic efficiency for thermal decomposition of the polymer backbone compared to the composite catalysts. However, since the azide groups exhibit a lower decomposition temperature, undergo exothermic decomposition, and generate nitrogen-containing compounds that catalyze the decomposition of other propellant components, this study specifically focuses on investigating the thermal decomposition behavior of the azide groups.

As shown in Figure 2c and Table 3, the temperature corresponding to the fastest weight loss of the azido group (*T*_p1_) in GAP-ETPE decreased upon the addition of catalysts, indicating that these catalysts exhibited certain catalytic effects on the thermal decomposition of the azido group. Among them, E-M showed only a 2.2 °C reduction in *T*_p1_, suggesting that MWCNTs had a weak catalytic effect on azido group decomposition. In contrast, E-F exhibited an 8.2 °C decrease in *T*_p1_, demonstrating that Fe_2_O_3_ possessed a moderate catalytic effect. The *T*_p1_ values of E-1 to E-8 were all lower than that of E-F, confirming that MWCNTs@Fe_2_O_3_ achieved the intended superior catalytic performance. From E-1 to E-8, *T*_p1_ initially decreased and then increased, with E-4 showing the lowest *T*_p1_. This indicates that the catalytic efficiency of MWCNTs@Fe_2_O_3_ was highest when the MWCNT content was 4%. This phenomenon can be attributed to the Fe-C synergistic catalytic effect, the adsorption capacity of the catalyst, and the number of active sites [20]. When a small amount of MWCNTs were incorporated into the gel framework of Fe_2_O_3_, the MWCNTs were insufficient to occupy all the pores of Fe_2_O_3_. As a result, the composite could adsorb a large amount of gas generated from azido group decomposition while providing sufficient active sites. As the MWCNT content increased, more C and Fe contributed to the synergistic catalytic effect, thereby enhancing the catalytic efficiency of the composite. However, when the MWCNT content further increased to 8%, excessive MWCNTs occupied all the pores of Fe_2_O_3_ (with some even remaining as surplus, as shown in Figure 1), leading to reduced gas adsorption capacity and fewer active catalytic sites, ultimately decreasing the catalytic efficiency. As illustrated in Figure 2d and Table 3, under the same material content conditions, E-1 exhibited a significantly lower *T*_p1_ than E-M+F, and the Δ*T* value of E-1 (representing its temperature difference from ETPE’s TP1) is more than double that of E-M+F, indicating that the MWCNTs@Fe_2_O_3_ composite had markedly superior catalytic performance compared to the mechanically mixed catalyst. This is because, in the composite, MWCNTs are embedded within the gel framework of Fe_2_O_3_, allowing C and Fe atoms to be in close proximity, thereby facilitating a stronger Fe-C synergistic catalytic effect. Interestingly, compared to E-F, the sample with 1% MWCNT addition, (E-M+F) showed a higher *T*_p1_. This can be attributed to the fact that the added MWCNTs existed as curled and agglomerated material (Figure 1), which hindered the effective contact between Fe_2_O_3_ and the sample to be catalyzed, thus diminishing the catalytic performance of Fe_2_O_3_.

Analysis of Figure 2e,f reveals that all samples exhibit only one exothermic peak, with the peak temperature corresponding to the weight loss temperature of the azido group in the TGA curves. This observation confirms that the decomposition of the azido group is an exothermic reaction. As shown in Table 3, the variation trend of the exothermic peak temperature (*T*_P_) across different samples aligns completely with that of *T*_p1_, for the reasons discussed earlier. Notably, the *T*_P_ of each sample is slightly lower than its corresponding *T*_p1_, which can be attributed to the uneven baseline of the DSC curves, introducing minor deviations in peak temperature determination. However, since the baseline trends are consistent across all samples, the calculated *T*_P_ variation trend remains accurate and does not affect the evaluation of the catalysts’ performance.

To further evaluate the catalytic performance of the MWCNTs@Fe_2_O_3_ composite, thermogravimetric analysis of GAP-ETPE was conducted at various heating rates to obtain TGA and DTG curves. Notably, all test samples exploded at 20 °C/min, and sample E-1 also exploded at 15 °C/min (see Supporting Information). These data were excluded from analysis since the energetic azido groups’ decomposition process could not be accurately captured during explosive events. Using the Kissinger method to analyze valid data, the calculated activation energies for azido group thermal decomposition were 105.4 kJ/mol (ETPE), 101.0 kJ/mol (E-F), and 92.9 kJ/mol (E-1). This progressive decrease in activation energy provides conclusive evidence for the exceptional catalytic performance of the MWCNTs@Fe_2_O_3_ composite.

In conclusion, the MWCNTs@Fe_2_O_3_ composite demonstrated significantly enhanced catalytic performance for the thermal decomposition of the azido group in GAP-ETPE, fulfilling the design objective.

### 3.3. Characteristics of GAP-ETPE-Based Propellants

**Figure 4:**
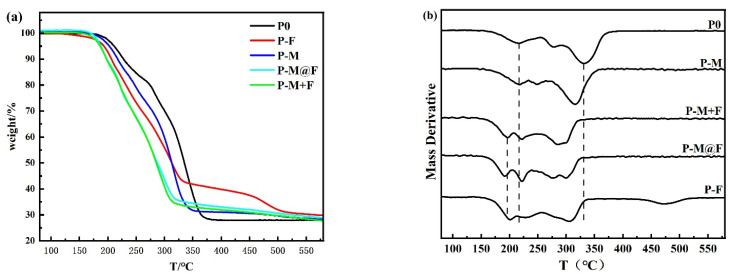
TGA (**a**) and DTG (**b**) curves of GAP-ETPE-based propellants.

This study aims to incorporate MWCNTs into the gel framework of Fe_2_O_3_ to achieve superior catalytic performance while mitigating potential adverse effects induced by MWCNT incorporation. As demonstrated in previous discussions, the MWCNTs@Fe_2_O_3_ composite exhibits outstanding catalytic activity for GAP-ETPE decomposition. Therefore, it is imperative to integrate this composite material into practical GAP-ETPE-based solid propellant formulations to validate its catalytic efficacy. The TGA and DTG curves characterizing the propellant’s thermal decomposition are presented in Figure 4.

As illustrated in Figure 4, the thermal decomposition of all propellant samples occurs through multiple distinct stages. Based on previous studies, these decomposition stages correspond sequentially from low to high temperature to the following: RDX decomposition, GAP-ETPE azido group degradation, AP (LTD), AP (HTD), GAP-ETPE hard segment breakdown, and GAP-ETPE backbone cleavage. Notably, certain decomposition stages exhibit overlapping behavior. Owing to the multicomponent nature of the propellant system, the thermal decomposition process demonstrates considerable complexity. For clarity, our analysis focuses primarily on comparative evaluation of the decomposition temperatures. The results reveal that the incorporation of catalysts leads to a systematic reduction in decomposition temperatures across all stages. Particularly, propellant sample P-M@F exhibits the lowest overall decomposition temperature profile.

These observations provide compelling evidence that the MWCNTs@Fe_2_O_3_ composite possesses superior catalytic performance compared to mechanically mixed counterparts, further validating its enhanced catalytic efficacy, while also confirming its substantial potential for practical applications. These results substantiate the practical significance of studying MWCNTs@Fe_2_O_3_ composites as catalysts for GAP-ETPE decomposition, while also establishing a clear research trajectory for our future work.

To evaluate whether the MWCNTs@Fe_2_O_3_ composite adversely affects other properties of GAP-ETPE-based propellants, we conducted comprehensive tests on tensile strength, elongation at break, and density, with the results summarized in Table 4.

As shown in Table 4, all four catalysts function as nano-reinforcing materials that enhance the propellant’s mechanical strength. In sample P-M, the high loading content of MWCNTs combined with shear forces during propellant processing leads to the formation of an interconnected MWCNT network within the propellant matrix, significantly improving its strength. However, due to the inherent rigidity of MWCNTs and their tendency to induce stress concentration at tube ends (as previously discussed), P-M exhibits poor elongation at break and reduced toughness. In contrast, P-M@F demonstrates optimal performance with both superior strength and toughness. This enhancement arises because: (1) nearly no exposed MWCNTs remain in the propellant matrix, and (2) Fe_2_O_3_ nanoparticles provide additional reinforcement. Particularly noteworthy is the marked performance advantage of P-M@F over the physically mixed P-M+F sample. Regarding density, which directly correlates with thrust per unit volume, two mechanisms contribute to its improvement: (1) nanoscale materials effectively fill microscopic pores in the solid propellant, and (2) the increased strength allows for higher compaction pressures during processing. As evidenced in Table 4, P-M@F achieves higher density than P0, reaching an advanced level among the tested formulations.

In summary, incorporating MWCNTs@Fe_2_O_3_ composites not only preserves but slightly enhances both the mechanical properties and density of GAP-ETPE-based propellants, confirming their practical suitability.

## 4. Conclusions

Employing reverse design principles, this study successfully synthesized MWCNTs@Fe_2_O_3_ composites by incorporating MWCNTs into the gel framework of Fe_2_O_3_, systematically investigating their catalytic effects on the thermal decomposition of both GAP-ETPE and GAP-ETPE-based solid propellants. Key findings demonstrate that the anchored MWCNTs@Fe_2_O_3_ composite exhibits superior catalytic performance compared to the following:

(i) Pristine Fe_2_O_3_ nanoparticles.
(ii) Unmodified MWCNTs.
(iii) Physically mixed MWCNTs+Fe_2_O_3_ counterparts.

The catalytic efficiency for azido group decomposition in GAP-ETPE shows clear composition dependence, reaching maximum activity at 4 wt% MWCNT loading. Notably, the composite achieves >2× enhancement in decomposition rate acceleration versus mechanically mixed catalysts at equivalent compositions. Crucially, the MWCNTs@Fe_2_O_3_ system maintains excellent compatibility with propellant formulations, inducing no detrimental effects on mechanical properties or other critical performance parameters.

This work establishes a novel methodology for the following:

- Rational design of hybrid nanocatalysts through structural confinement;
- Performance optimization via composition–structure relationships;
- Practical implementation of nanocarbons in next-generation energetic materials.

## Figures and Tables

**Table 1:** The nomenclature of different samples.

GAP-ETPE	Catalyst	Catalyst Composition
ETPE	-	-
E-F	F	Fe_2_O_3_
E-1	M@F-1	MWCNTs@Fe_2_O_3_ (MWCNTs content: 1 wt%)
E-2	M@F-2	MWCNTs@Fe_2_O_3_ (MWCNTs content: 2 wt%)
E-4	M@F-4	MWCNTs@Fe_2_O_3_ (MWCNTs content: 4 wt%)
E-8	M@F-8	MWCNTs@Fe_2_O_3_ (MWCNTs content: 8 wt%)
E-M	M	MWCNTs
E-M+F	M+F	MWCNTs (1 wt%), Fe_2_O_3_ (99 wt%)

**Table 2:** The designations and preparation information of the propellants (mass percentage).

Propellant	GAP-ETPE	RDX	Al	AP	Catalyst			
					MWCNTs@Fe_2_O_3_	MWCNTs	Fe_2_O_3_	Notes
P0	20	12	20	48	0	0	0	
P-M	20	11.2	19.2	47.2	0	2.4	0	M
P-M+F	20	11.2	19.2	47.2	0	0.024	2.376	M+F
P-M@F	20	11.2	19.2	47.2	2.4	0	0	M@F-1
P-F	20	11.2	19.2	47.2	0	0	2.4	F

**Table 3:** TGA and DSC parameters of MWCNTs@Fe_2_O_3_/GAP-ETPE.

Samples	*T*_P1_ (°C)	Δ*T* (°C)	*T*_P_ (°C)
ETPE	252.3	0	248.9
E-M	250.1	2.2	247.5
E-8	243.2	9.1	241.2
E-4	239.6	12.7	236.9
E-2	242.7	9.6	241.1
E-1	243.2	9.1	242.1
E-F	244.1	8.2	242.5
E-M+F	248.2	4.1	246.8

**Table 4:** Other Properties of GAP-ETPE-Based Propellants.

Samples	*σ_m_* (MPa)	*ε_b_* (%)	*Ρ* (g/cm^3^)
P0	3.46	14.7	1.69
P-M	8.22	12.7	1.81
P-M+F	3.88	21.1	1.81
P-M@F	4.95	21.6	1.77
P-F	3.83	14.8	1.73

## Data Availability

The original contributions presented in this study are included in the article/Supplementary Material. Further inquiries can be directed to the corresponding authors.

## Supplementary Materials

The following supporting information can be downloaded at: https://www.mdpi.com/article/10.3390/polym17152080/s1, Figure S1. TGA curves of GAP-ETPE with carbon-based catalysts. Figure S2. DTG curves of GAP-ETPE with carbon-based catalysts. Figure S3. TGA curves of AP with carbon-based catalysts. Figure S4. DTG curves of AP with carbon-based catalysts. Figure S5. X-ray diffraction (XRD) patterns for various samples. Figure S6. TGA (left) and DTG (right) curves of ETPE. Figure S7. TGA (left) and DTG (right) curves of E-F. Figure S8. TGA (left) and DTG (right) curves of E-1.
