# Supplementary material for: Preparation of Inverse-Loaded MWCNTs@Fe2O3 Composites and Their Impact on Glycidyl Azide Polymer-Based Energetic Thermoplastic Elastomer
Source: Polymers (Basel). 2025 Jul 30;17(15):2080. doi: 10.3390/polym17152080 (PMC12349281; doi:10.3390/polym17152080)
Supplement: Supplementary file 1 [file polymers-17-02080-s001.zip › polymers-3712315-supplementary.pdf]

Supplementary Material

Preparation of Inverse-Loaded MWCNTs@Fe_2O_3 Composites and Their Impact on Glycidyl Azide Polymer-Based Energetic Thermoplastic Elastomer

Shuo Pang [1,2,3], Yihao Lv [1,2,3], Shuxia Liu [1,2,3], Chao Sang [1,2,3,*], Bixin Jin [4] and Yunjun Luo [4,*]

[1] Shandong Provincial Key Laboratory of Monocrystalline Silicon Semiconductor Materials and Technology, Dezhou University, Dezhou 253023, China
[2] School of Chemistry and Chemical Engineering, Dezhou University, Dezhou 253023, China
[3] Shandong Provincial Engineering Research Center of Organic Functional Materials and Green Low-Carbon Technology, Dezhou 253023, China
[4] School of Materials Science and Technology, Beijing Institute of Technology, Beijing 100086, China
* Correspondence: 18810988921@163.com (C.S.); yjluo@bit.edu.cn (Y.L.)

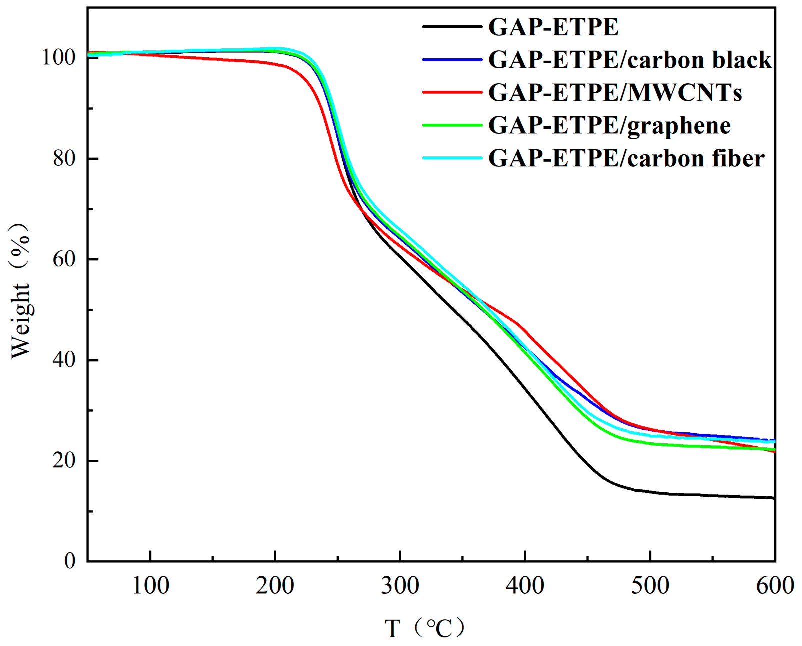

Figure S1. TGA curves of GAP-ETPE with carbon-based catalysts.

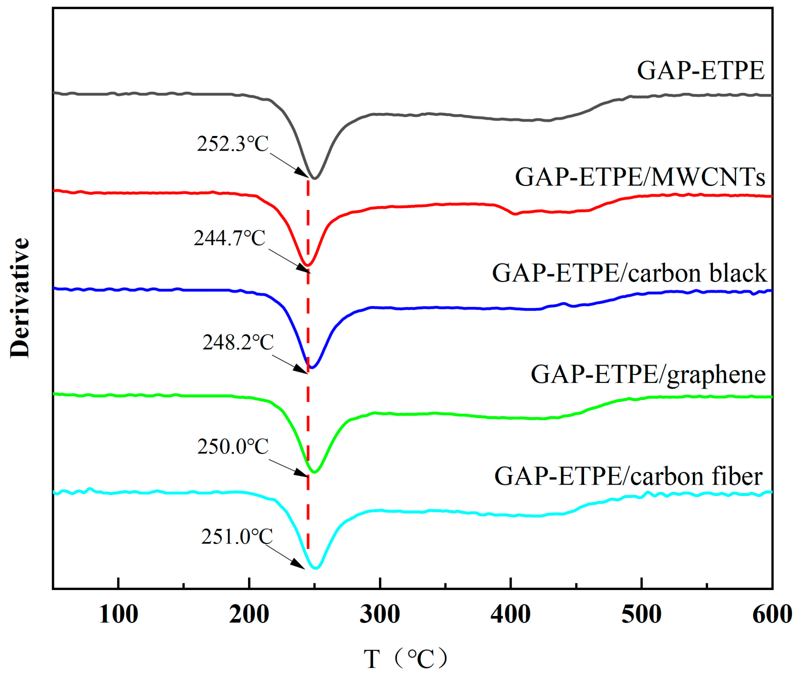

Figure S2. DTG curves of GAP-ETPE with carbon-based catalysts.

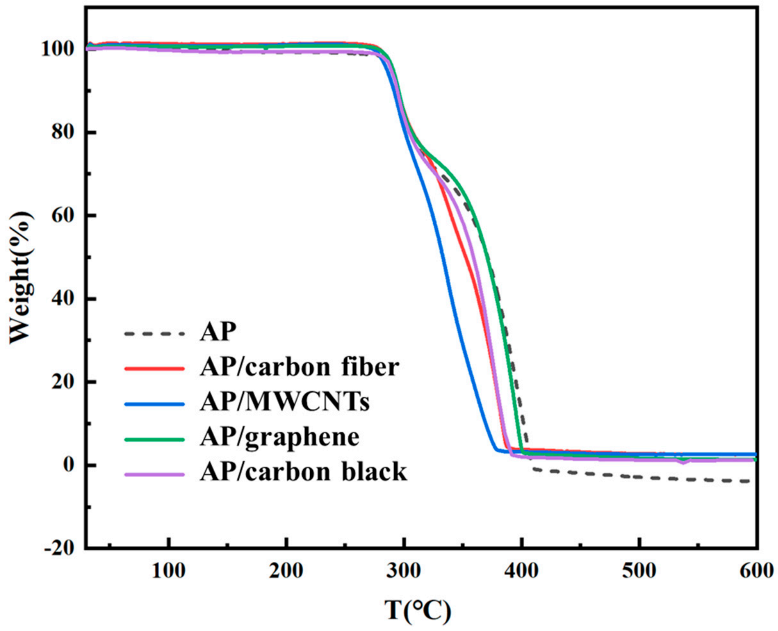

Figure S3. TGA curves of AP with carbon-based catalysts.

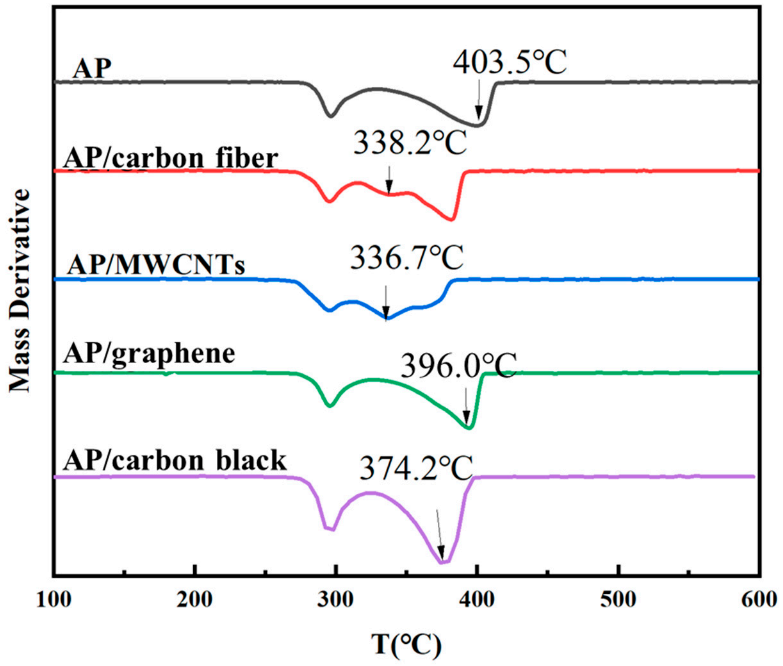

Figure S4. DTG curves of AP with carbon-based catalysts.

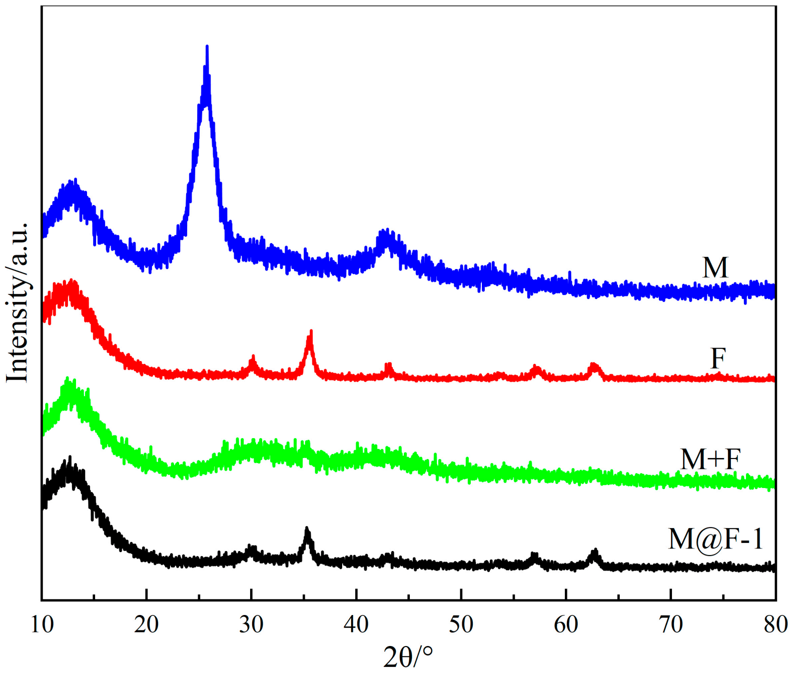

Figure S5. X-ray diffraction (XRD) patterns for various samples.

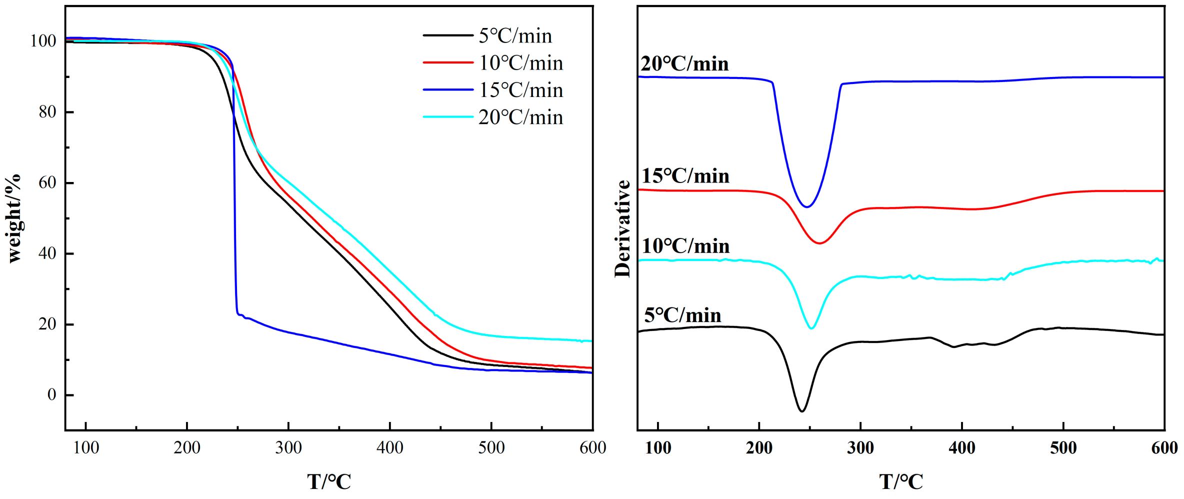

Figure S6. TGA (left) and DTG (right) curves of ETPE.

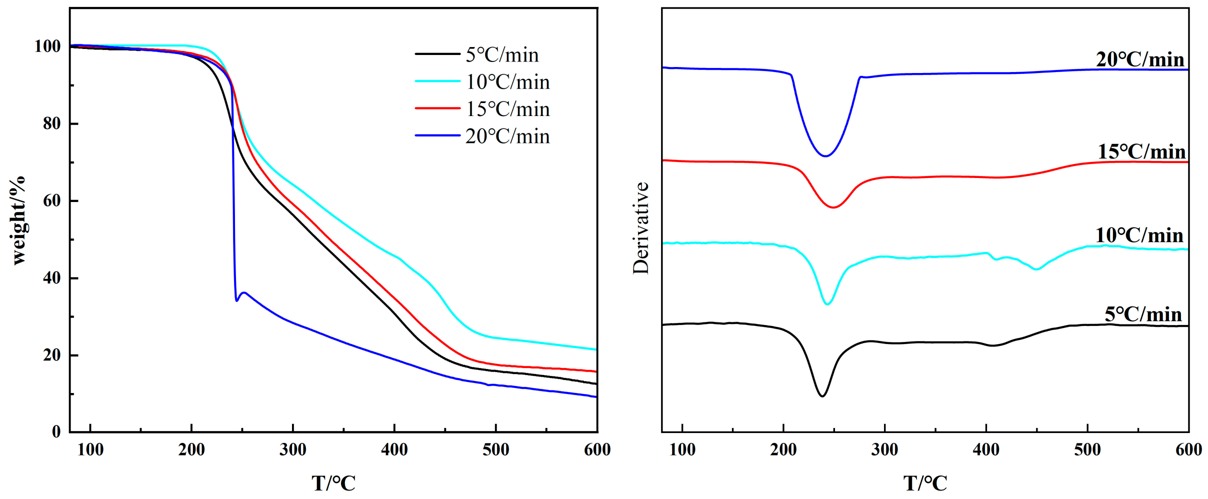

Figure S7. TGA (left) and DTG (right) curves of E-F.

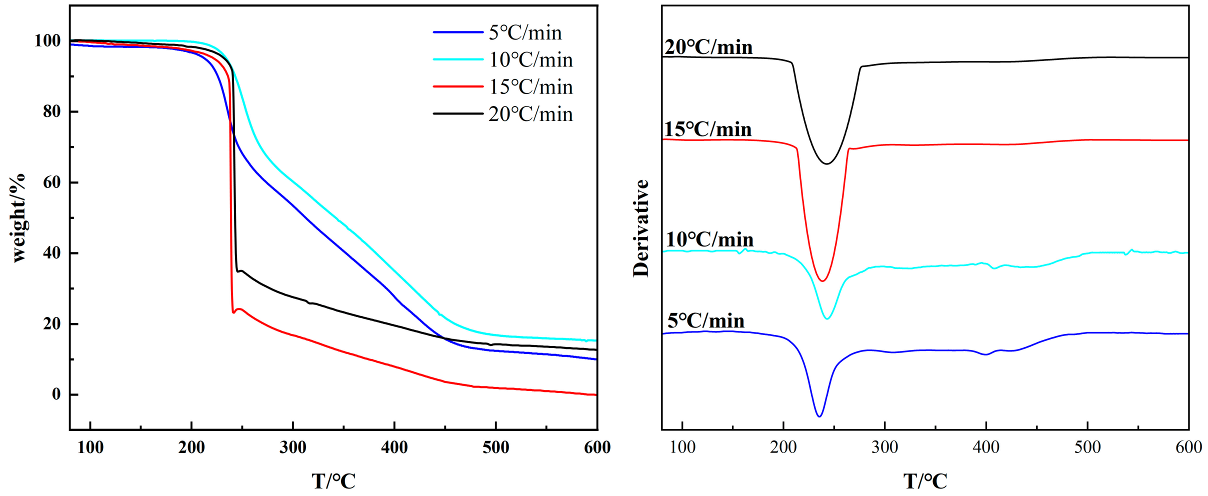

Figure S8. TGA (left) and DTG (right) curves of E-1.
